# What is genomic medicine?

**DOI:** 10.5195/jmla.2019.604

**Published:** 2019-07-01

**Authors:** Stephanie Clare Roth

**Affiliations:** Biomedical and Research Services Librarian, Ginsburg Health Sciences Library, Temple University, Philadelphia, PA 19140, stephanie.roth@temple.edu

## Abstract

Genomic medicine is rapidly changing the future of medicine. Medical librarians need to understand this field of research and keep current with its latest advancements. Even if they are not directly involved in genomic medicine, librarians can play an integral role by helping health care consumers and practitioners who may also need to expand their knowledge in this area. This article provides a basic introduction to genomic medicine, gives a brief overview of its recent advancements, and briefly describes some of the ethical, legal, and social implications of this emerging area of research and practice.

## BACKGROUND

Genomic medicine is advancing very quickly, so rapidly that some health care providers lack understanding of the ethical, legal, and social implications that can affect how they practice medicine and share information with their patients in the future. Librarians can play a vital role in bridging this knowledge gap.

Unfamiliarity with genomic medicine can create communication barriers between scientists and nonscientists, as the general public and even general health care practitioners might not sufficiently understand genomic medicine. Health care practitioners will need to be trained to recognize how the genome can impact a person’s health and to understand the magnitude of the ethical decisions and the implications of those decisions that they must be ready to face in the future. As in any rapidly changing field of medicine, there is a need for more evidence-based research in genomic medicine, where the clinical evidence currently lags behind the science [1]. With the rapid rise of mainstream precision medicine and genetic testing, many health care providers might not be ready to dive into interpreting genetic tests or communicating test results and providing help with decision-making for their patients. This has led to growth in genetic counseling, a medical field in which professionals with genetics backgrounds are able to weigh the needs of their patients and the impact of genetic results on the patients’ family members to promote “autonomous decision making” [2].

Librarians—whether public or medical—may be called upon to understand genomic medicine in order to provide relevant resources for health care providers and can play an important role in consumer education. Some medical librarians have expertise in genomic medicine or bioinformatics and offer workshops, instruction, services, or library guides related to genomic medicine. However, before nonexpert librarians can provide services related to genomic medicine, they must be ready to expand their basic understanding of its concepts and gain awareness of its ethical, legal, and social implications.

As genomic medicine has expanded beyond the clinic and is now in the hands of the consumer, the need for librarians to become knowledgeable in this area has expanded beyond hospital and even medical libraries. Public librarians are now providing consumer education and outreach related to precision medicine and are creating opportunities to engage citizen scientists with support from the National Network of Libraries of Medicine (NNLM) [3]. With the plethora of news, social media, and marketing around genomic medicine, librarians are needed now more than ever to continue their role in providing authoritative sources, rigorous searches, bioinformatics training, assistance with data or data visualization, and consumer health information. With many examples of librarians paving the way in the field of genomic medicine [4–7], librarians may be expected to increase their engagement with local communities and their provision of resources for citizen scientists.

## WHAT IS GENOMIC MEDICINE?

Genomic medicine is an interdisciplinary medical specialty involving the use of genomic information that has rapidly grown since the completion of the Human Genome Project (HGP) more than a decade ago. Definitions of basic concepts of genomic medicine are provided in Table 1.

**Table 1:** Basic concepts in genomic medicine

Term	Description
Biomarker	Also called a biological marker, a classical biomarker is any characteristic that can be used to assess a particular disease state or physiological function. A genomic biomarker is “a DNA or RNA characteristic that is an indicator of normal biologic processes, pathogenic processes, and/or response to therapeutic or other intervention” [8]. A genomic biomarker reflects the expression, function, or regulation of a gene.
Codon	DNA or RNA nucleotides or bases are read in groups of three (e.g., ATG, AUG), which are called codons. Start and stop codons show when a protein sequence starts or ends.
DNA	Deoxyribonucleic acid (DNA) is the carrier of genetic information. DNA consists of four nucleotides or bases (A, T, G, and C). DNA can replicate or make copies of itself.
Exome	The approximately 1% of the genome formed only by exons.
Exon	The protein coding sequence of DNA (the part of the genome that is expressed).
Gene	A gene is a specified sequence of DNA that serves as the basic unit of heredity [9]. “Gene” comes from the Greek word *genea*, meaning generation.
Gene expression	When a gene is turned on and its RNA or protein product is being made, the gene is said to be expressed. The on/off state of cells is called a gene expression profile, with each cell type having a unique profile [10].
Genome	The genome includes all of an organism’s DNA, including both exons and introns.
Germline	Germline cells are sperm, egg, or embryo cells. Changes to the germline are permanent. Germline traits or mutations are inherited and generational.
Intron	The non-protein coding sequence of DNA (the part of the genome that is not expressed).
MicroRNA	MicroRNA (miRNA) is a type of genetic material that regulates gene expression. miRNAs are promising biomarkers and can point toward development of new therapeutic approaches.
RNA	Ribonucleic acid (RNA) is a single-stranded copy of the DNA sequence that plays a messenger role to help cells carry out instructions for making a protein. RNA consists of four nucleotides or bases (A, U, G, and C). The DNA T is replaced by the RNA U when copied.
SNP	A single-nucleotide polymorphism (SNP, pronounced “snip”) is a DNA sequence variation that occurs when a single nucleotide (A, T, C, or G) in a gene sequence is altered. SNPs are the most abundant variant in the human genome and are the most common source of genetic variation, with more than 10 million SNPs present in the human genome. They can also serve as biomarkers.
Somatic	Somatic cells include stem cells, blood cells, and other cell types. Changes to somatic cells are not permanent, meaning they cannot be passed down by generation. Somatic cell mutations include acquired alterations that can result from chemical or radiation exposure. Changes may also occur as cells are copied during growth or repair processes.

The genome is the complete set of information in an organism’s DNA [11]. A DNA molecule consists of two long chains or strands. Each DNA chain contains a genetic code written using only four letters, which represent nucleotide subunits or bases: adenine (A), guanine (G), thymine (T), and cytosine (C). Their order determines the instructions contained in a strand of DNA [12]. Each DNA chain is twisted to form a double helix [11]. The 1953 discovery that two molecules paired to form the double helix earned James Watson, Francis Crick, and Maurice Wilkins a Nobel Prize. This discovery was made possible by an -ray image of DNA produced by Rosalind Franklin, who, although not formally honored with the Nobel Prize, remains an important figure in the history of genomic medicine [13]. To carry out instructions for making proteins, which are essential for the function of every cell in the body, a sequence of the DNA molecule, or gene, is copied into multiple single-stranded ribonucleic acid (RNA) molecules. A new sequence in RNA is formed in which the DNA T molecule is replaced with the RNA uracil (U) molecule [14].

Humans are estimated to have between 20,000 and 25,000 genes [15], which are the smallest units of heredity. Humans have two copies of each gene, inheriting one from each parent. The human genome contains both protein-coding genes and non-protein-coding genes. In humans, genes may consist of only a few hundred or more than 2 million DNA bases [15]. Interestingly, humans have just a few more genes than worms and fewer genes than rice or wheat. Thus, the genome reflects not only gene count, but also the complexity of gene networks [16]. To quote Mukherjee, “The human genome is fiercely inventive, it is dynamic, parts of it are surprisingly beautiful, it is encrusted with history, it is inscrutable, vulnerable, resilient, adaptable, repetitive, and unique. It is poised to evolve. It is littered with debris of its past. It is destined to survive. It resembles us” [16].

Completed in 2003, the HGP was a thirteen-year project coordinated by the US Department of Energy and the National Institutes of Health to sequence the complete human genome from volunteers who provided DNA samples. Knowing privacy was a concern, it is intentionally not known who volunteered. All labels were removed before samples were chosen, and volunteers gave informed consent before participating in the project. Although this project is finished, analyses of its data continue. HGP and related data are available through National Center for Biotechnology Information (NCBI) databases [17], and NNLM offers a course for librarians who are interested in learning how to search them [18].

## ADVANCES IN GENOMIC MEDICINE

Several noteworthy advances or achievements in genomic medicine are described below. However, more in-depth study of these topics beyond that provided in these brief summaries is warranted.

### Precision medicine

The end goal of precision medicine is that instead of a “one size fits all” approach by disease type, medicine will be informed by a genetic understanding of the disease. Precision medicine not only involves studying the genome, but also considers factors like where a person lives, what they do, and what their family health history is [19]. The goal is to develop targeted prevention or treatment approaches to help specific individuals stay healthy or get better instead of relying on approaches that are the same for everyone. In 2018, the largest precision medicine research study in the United States, All of Us, launched with a goal of recruiting one million participants [20].

### CRISPR

Clustered Regularly Interspaced Short Palindromic Repeats (CRISPR) was originally discovered by yogurt engineers Philippe Horvath and Rodolphe Barrangou of Danisco (DuPont) in their study of the sequences of the bacterial genome that protect against invading viruses [21]. In 2012, RNA biologists Jennifer Doudna and Emmanuelle Charpentier used their knowledge of CRISPR to reprogram DNA using an enzyme called CRISPR-associated protein 9 (Cas9), which is described as programmable “molecular scissors” that can rewrite the genetic code [22].

A commonly used analogy is to think of CRISPR Cas9 as a text editor for the genetic code, with the ability to cut out or permanently replace DNA. With the ability to cut out and replace the “bad” DNA with “good” DNA, CRISPR Cas9 offers considerable promise to cure several cancers, solid tumors, melanoma, leukemia, HIV, β-thalassemia, sickle-cell anemia, and other diseases. Currently, clinical applications of somatic (i.e., nonreproductive) cell CRISPR gene editing are allowed in humans, which do not pass down heritable changes to future generations, and CRISPR Cas9 is important for drug discovery. However, it is currently unethical and banned in the United States and most other countries to genetically alter the DNA of human embryos used for implantation, as changes to germline cells are permanent and the implications of these changes are currently unknown. Another fear is that CRISPR Cas9 can open up the door to questionable “designer baby” applications aimed to improve physical appearance or perform other enhancements.

### Omics

In addition to genomics and the study of genes, “omics” is a general term used to describe the many areas of study related to genomics that focus on other biological processes of a living organism. These may include, but are not limited to, pharmacogenomics (i.e., role of the genome in drug response), epigenomics (e.g., methylation and histone alterations of the genome), proteomics (i.e., the complete set of proteins in cells), transcriptomics (i.e., the complete set of RNA transcripts in cells), metabolomics (i.e., collective study of small molecules), and metabonomics (i.e., study of metabolic processes) [23].

### Genetic testing

Two classes of genetic testing are now available: clinical or direct-to-consumer (DTC). A clinical genetic test is usually done in a clinical environment with access to trained medical professionals, such as genetic counselors, to help patients interpret the results, which can be very easy to misinterpret. For example, sometimes the results of a genetic test can give a false sense of assurance or sound a false alarm, but a conversation with a genetic counselor can help put the test results into context or explain their implications.

By contrast, DTC genetic testing is done at home after ordering a simple test kit online. Examples of DTC genetic test kits are 23andMe, AncestryDNA, National Geographic Geno 2.0, My Heritage DNA, Habit, Pheramor, and DNAFit. However, the validity of some DTC genetic tests is questionable [24], and their results are not usually interpreted by a qualified medical professional; thus, their purpose may be more recreational than medical. Privacy and security of genetic data are not completely guaranteed with any DTC genetic test, but consumers can take control of their privacy by knowing and reading the privacy policy and opting out of consent so that their information is not shared with a third party.

### Gene therapy

Gene therapy is a type of treatment in which healthy foreign genetic material is inserted into a person’s cells to cure a rare condition or disease [25]. Instead of only treating symptoms, gene therapy aims to correct the underlying genetic cause of the disease and, thus, serve as a one-shot cure. Gene therapy can be applied to cancer immunotherapy through the use of chimeric antigen receptor (CAR) T-cell therapy with one’s own immune cells to find and destroy cancer cells. CRISPR Cas9 has enabled next-generation CAR T-cells to enhance the therapeutic potential of CAR-T cell treatments [26].

In 2017, the Food and Drug Administration (FDA) approved the first CAR T-cell-based gene therapy in the United States. Known as Kymriah (tisagenlecleucel), this is a genetically modified T-cell immunotherapy for treating patients who are children or young adults with a rare form of acute lymphoblastic leukemia [27]. The second FDA-approved CAR-T cell-based gene therapy, also in 2017, is Yescarta (axicabtagene ciloleucel), which is for adult patients with certain types of non-Hodgkin’s lymphoma [28]. While gene therapies are becoming available, they are not yet affordable to the general public. Kymriah is priced at $475,000, and Luxturna, which is used to treat a rare form of vision loss, has a list price of $850,000.

## ETHICAL, LEGAL, AND SOCIAL IMPLICATIONS

### Concerning gene editing

The gene editing of human embryos is currently not allowed by the United States in publicly funded research [29] and is completely banned in many countries. In both China and the United Kingdom, researchers can conduct CRISPR experiments of in-vitro human embryos for a few days after fertilization [30], but their implantation in the uterus is not allowed. These three countries cohost an International Summit on Human Gene Editing for experts to discuss relevant ethical issues. The second summit was held in November 2018 in Hong Kong [31]. At this summit, to the shock and outrage of the scientific community, the Chinese scientist He Jiankui announced he had successfully delivered the first CRISPR gene-edited babies, who were twins. Now more than ever, the fear of “designer babies” is in the tangible future [32]. Some concerns around gene editing include:

- creation of “designer babies” with nonmedical enhancements (e.g., height, eye or hair color, intelligence, athletic ability)
- false distinction between traits that are “normal” versus those considered a disability or disorder
- permanent and hereditary changes to the germline that cannot be reversed
- uncertainty regarding whether CRISPR is safe or effective in humans
- unknown consequences of removing or editing a DNA sequence

### Concerning gene therapy

Gene therapy has been controversial, and research came to a halt in the United States in 1999 after eighteen-year-old Jesse Gelsinger died from gene therapy complications [33]. With advances in technology, however, gene therapy is making a strong comeback. In 2018, the FDA approved the first gene therapy in the United States and announced that there will be less oversight for gene therapies [34]. Some concerns around gene therapy include:

- false new confidence in its safety or efficacy
- high financial cost, which makes it less accessible to those in need of treatment
- existence of amateur scientists who self-inject with gene therapeutic agents (i.e., “garage geneticists”) without a qualified health professional

### Concerning direct-to-consumer genetic testing

DTC genetic tests are increasing in popularity because they do not need to be ordered by a doctor or approved by insurance; the test kit is mailed directly to one’s doorstep; and results can be reviewed privately. Although they are fast and convenient, a Federal Trade Commission consumer alert states that some DTC genetic tests lack scientific validity, and consumers are cautioned to be skeptical of claims [24]. The marketing tactics and privacy policies of some DTC genetic tests are also cause for concern. Consumers should be aware of the following aspects of DTC genetic testing:

- data privacy or breaches
- primarily entertainment rather than medical purposes (e.g., Pheramor for dating compatibility)
- public misunderstanding of informed consent or privacy policies
- test results that are difficult to interpret without a genetic counselor or trained medical professional
- consumers’ lack of realization about the limitations of DTC genetic testing (absence of a medical professional to interpret results, difficulty in determining disease risk, or overestimation of the genetic significance)
- consumers’ difficulty in taking primary ownership of their genetic data after they have been shared with companies
- double dipping of company profits with every sale (i.e., profiting from both the sale of the test and the sale of the test results data to research partners such as pharmaceutical companies), unless consumers opt out: “By buying into 23andMe you are not a consumer or user, you are in fact the product” [35]

## CONCLUSION

By becoming familiar with genomic medicine, librarians will be able to better serve the needs of their users in this time of rapid medical advancement and development. Librarians can also serve as community partners, create relevant programming, and better understand the implications of this emerging interdisciplinary area of research. Some librarians are already paving the way forward, such as using an artificial intelligence (AI) chatbot known as Plutchik to engage younger learners in searching across the NCBI suite of databases [4] and administering a survey to identify bioinformatics training topics of interest to health sciences researchers [5]. In addition, a librarian published a paper informing hospital librarians of what they need to know to support researchers as medicine shifts from one-size-fits-all to precision medicine, including examples of relevant search requests with results [6]. Medical librarians are also incorporating experiential learning in data visualization into their medical education to prepare students for data-driven decision-making and to provide them with the skills they need to analyze big data sets, an important aspect of genomic or precision medicine [7]. These are just a few of many ways in which librarians are already expanding their roles in genomic medicine.
